# A Case Report of Spinal Dural Arteriovenous Fistula: A Threatening Cause of Paraplegia

**DOI:** 10.7759/cureus.31469

**Published:** 2022-11-14

**Authors:** Nizar El Bouardi, Y. Lamrani, Meriem Haloua, Badreddine Alami, Meryem Boubbou, Mustapha Maaroufi

**Affiliations:** 1 Faculty of Medicine and Pharmacy, Sidi Mohamed Ben Abdellah University, Fez, MAR; 2 Radiology, Hassan II University Hospital of Fez, Fez, MAR

**Keywords:** angiography, s: magnetic resonance imaging, myelopathy, paraplegia, dural arteriovenous fistula, spinal cord

## Abstract

Spinal dural arteriovenous fistulas (SDAVFs) are rare entities and are often misdiagnosed. They usually occur in adults above the age of 50 and 60 years. While they most commonly involve the thoracolumbar region, they can occur anywhere along the spinal cord. Clinical symptoms are insidious and not specific and may progress slowly, over several years, to severe myelopathy with paraplegia. Early diagnosis is critical because the deficits are potentially reversible if carefully treated. Delayed treatment may result in severe and irreversible neurological disability. Imaging diagnosis relies on MRI and conventional spinal angiography. Once identified, the dural arteriovenous fistula should be immediately treated by either endovascular embolization or surgical ligation. In this report, we present a case of SDAVF in a 65-year-old male that was managed by open surgery.

## Introduction

Vascular malformations of the spine are very rare, accounting for only 2% of vascular neurological pathologies [1]. They are classified into arteriovenous fistulas and arteriovenous malformations. Spinal dural arteriovenous fistulas (SDAVFs) are consistently the most common type, representing up to 80% of lesions identified [2]. They are rare and often misdiagnosed and occur predominantly in men over the age of 50 and 60 years and lead to chronic myelopathy due to venous hypertension [2]. If untreated, SDAVF may evolve slowly into permanent paraplegia. Based on a case report and review of the literature, we discuss its pathophysiology, clinical presentations, imaging appearances, and treatment modalities.

## Case presentation

**Figure 1:**
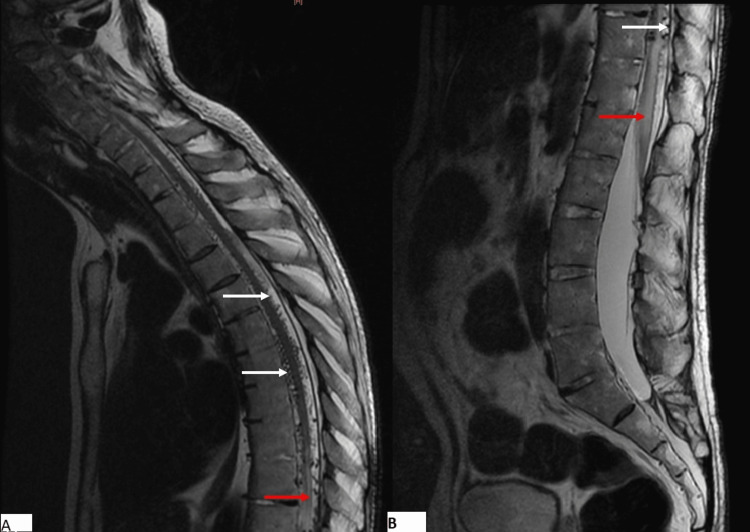
Sagittal T2WI of the cervicothoracic (A) and lumbar (B) spine showing hyperintense enlarged spinal cord and conus medullaris (red arrow), associated with flow void (white arrows) corresponding to enlarged veins

A 65-year-old male, with a remarkable medical history involving poorly managed diabetes mellitus and hypertension, was admitted for the evaluation of progressive paraplegia for 18 months. This was associated with tingling in the lower extremities. On admission, his blood pressure was 170/90 mmHg, and his heart rate was normal at 75 pulses per minute. He was alert and oriented but had difficulty standing up. Physical examination revealed a complete impairment of muscle function in both lower extremities. Osteotendinous reflexes were polykinetic and the Babinski sign was positive in both feet. Sensory perceptions of pain, vibration, and touch were impaired. Based on these findings, we graded his condition as Frankel grade B. We performed an MRI of the spine and brain, which revealed no compression but a tumefied thoracolumbar spinal cord, revealing a high signal intensity in T2-weighted images (Figure 1) and low signal intensity in T1-weighted images. MRI findings also showed prominent serpentine T2 flow voids in the intradural space corresponding to dilated perimedullary veins (Figure 1).

**Figure 2:**
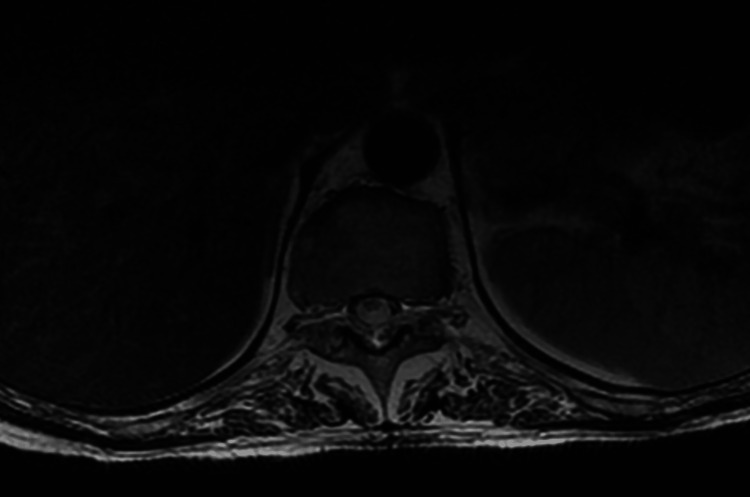
Axial T2WI of the spine showing the similar involvement of the white and gray matter of the spine

Axially, the abnormality involved both gray and white matter (Figure 2).

**Figure 3:**
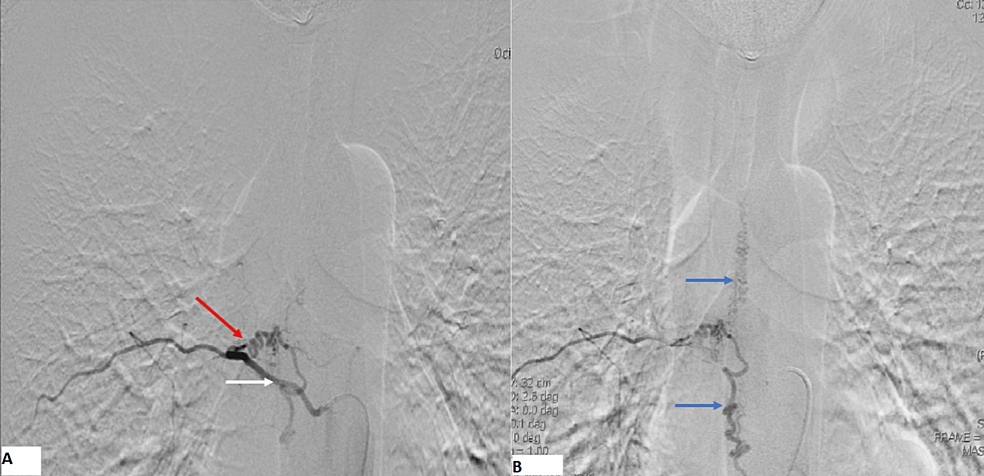
Angiographic view showing the shunt (red arrow) between a radiculomeningeal artery emanating from the 8th right intercostal artery (white arrow) and radiculomedullary vein, associated with dilated and tortuous perimedullary veins (blue arrows)

**Figure 4:**
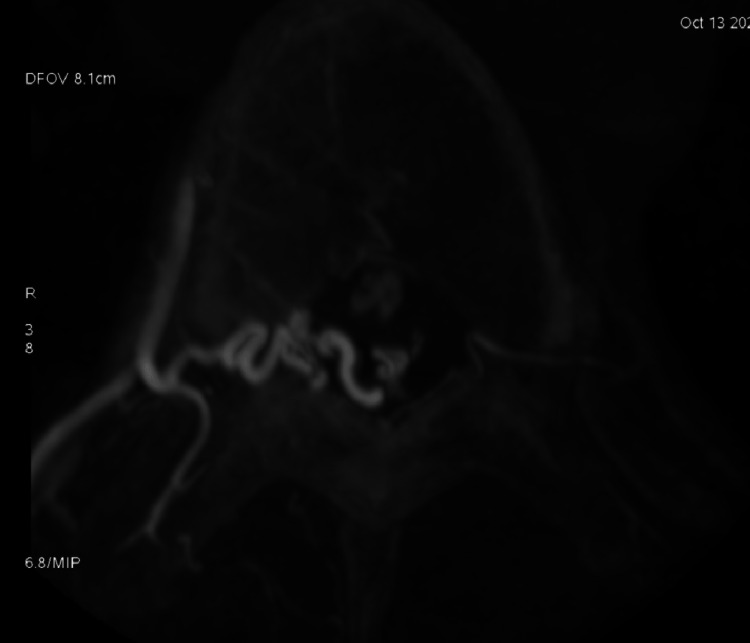
3D angiographic view showing the shunt within the nerve root

Based on this finding, congestive myelopathy complicated by SDAVF was suspected. A spinal angiography was performed, which showed an intradural shunt between a radiculomeningeal artery emanating from the 8th right intercostal artery and radiculomedullary veins, associated with dilated and tortuous perimedullary veins (Figures 3, 4). We also noted a retrograde contrast uptake within radiculomedullary veins.

After a multidisciplinary consultation involving neurologists, neurosurgeons, and interventional radiologists, the case was managed by open surgery. Subsequent follow-up visits and clinical examination showed a partial recuperation of motor and sensory functions.

## Discussion

SDAVF is a rare pathology but remains the most common vascular shunt of the spine; it is characterized by abnormal communication between arteries (branches of the radiculomeningeal artery) and veins (a radicular vein) [3]. These connections are classically located within the dura mater near spinal nerve roots. While its etiopathology remains unclear, these are presumed to be acquired lesions. Some potential predisposing factors have been described, including thrombosis of the extradural spinal veins and traumatic injury, although some lesions may be idiopathic [3,4]. The condition is most commonly found in males in their early 60s. The average duration of the onset is usually four weeks [1]. They occur at the thoracic level, frequently at the T7 level [1].

The typical clinical presentation is progressive myelopathy associated with motor disorders (up to 78% at the time of diagnosis) most often involving the lower limbs, accompanied by sensory (up to 69%) and sphincter disorders (up to 80%), and usually micturition disorders [3]. Up to 16% of patients also present with unilateral symptoms [3]. Symptoms typically worsen with Valsalva-type activities and improve with rest [5]. If left untreated, it evolves into permanent paraplegia. Cases of acute aggravation have also been reported [5].

Radiological diagnosis of SDAVF is based on MRI and conventional angiography. The main findings on MRI that suggest the possibility of SDAVF include swelling of the spinal cord with corresponding T2 hyperintensity and T1 hypointensity, predominantly affecting the lower thoracic region and the conus. The margins are often described as "flame-shaped" [6]. Both gray and white matter are affected. Areas of signal change and cord enlargement do not necessarily correspond to the fistula location [7]. Spinal cord parenchyma may enhance after intravenous gadolinium injection. All of these signs are not specific and many differentials can be considered, including transverse myelitis, spinal stenosis, and spinal infarction.

The most specific MRI findings are as follows:

- Enlarged vessels on the surface of the cord, which is only found in approximately 50% of patients with SDAVF. Multisegment hyperintensities with associated subarachnoid flow voids are pathognomonic of SDAVF [8].

- A heterogeneous enhancement showing some regions of non-enhancement describing the missing-piece sign [6].

Spinal angiography remains the gold standard for diagnosis and confirmation. Typical findings are contrast stasis within radiculomedullary arteries, delayed venous return following injection, and retrograde contrast uptake within radiculomedullary veins [6].

Treatment of SDAVF usually involves endovascular embolization (using biological glue or Onyx™) or open surgery. Embolization is typically the preferred option for extradural arteriovenous fistulas, whereas the treatment of intradural fistulae is performed via either embolization or open surgery [9]. Open surgery is more commonly preferred and offers reduced recurrence and morbidity as well as improved success rates compared with embolization.

Conservative management may be appropriate for asymptomatic SDAVF but should be carried out under close clinical and imaging surveillance. The most suitable imaging modality is MRI, which can reveal features of developing edema myelopathy. Once clinical or radiological symptoms appear, appropriate treatment should be implemented to stop the evolution and improve functional outcomes.

## Conclusions

This case report illustrated a rare cause of progressive paraplegia. When encountering cases of a tumefied spinal cord associated with perimedullary flow voids, a diagnosis of SDAVF should be considered and confirmed by conventional angiography. Thus, radiologists should be familiar with the imaging features of SDAVF so that unnecessary delays in diagnosis and treatment can be avoided, as well as further invasive testing for pathologies with similar presenting symptoms.
